# Financial barriers and coping strategies: a qualitative study of accessing multidrug-resistant tuberculosis and tuberculosis care in Yunnan, China

**DOI:** 10.1186/s12889-017-4089-y

**Published:** 2017-02-22

**Authors:** C. Hutchison, M. S. Khan, J. Yoong, X. Lin, R. J. Coker

**Affiliations:** 1 London School of Hygiene and Tropical Medicine, London, UK; 2 Saw Swee Hock School of Public Health, National University of Singapore, Singapore, Singapore; 3 Centre for Economic and Social Research, University of Southern California, Los Angeles, USA; 4 Yunnan Center for Disease Control and Prevention, Kunming, China; 5 Faculty of Public Health, Mahidol University, Bangkok, Thailand

**Keywords:** Tuberculosis, Multidrug-resistance, China, Barriers, Qualitative

## Abstract

**Background:**

Tuberculosis (TB) and multidrug-resistance tuberculosis (MDR-TB) pose serious challenges to global health, particularly in China, which has the second highest case burden in the world. Disparities in access to care for the poorest, rural TB patients may be exacerbated for MDR-TB patients, although this has not been investigated widely. We examine whether certain patient groups experience different barriers to accessing TB services, whether there are added challenges for patients with MDR-TB, and how patients and health providers cope in Yunnan, a mountainous province in China with a largely rural population and high TB burden.

**Methods:**

Using a qualitative study design, we conducted five focus group discussions and 47 in-depth interviews with purposively sampled TB and MDR-TB patients and healthcare providers in Mandarin, between August 2014 and May 2015. Field-notes and interview transcripts were analysed via a combination of open and thematic coding.

**Results:**

Patients and healthcare providers consistently cited financial constraints as the most common barriers to accessing care. Rural residents, farmers and ethnic minorities were the most vulnerable to these barriers, and patients with MDR-TB reported a higher financial burden owing to the centralisation and longer duration of treatment. Support in the form of free or subsidised treatment and medical insurance, was deemed essential but inadequate for alleviating financial barriers to patients. Most patients coped by selling their assets or borrowing money from family members, which often strained relationships. Notably, some healthcare providers themselves reported making financial and other contributions to assist patients, but recognised these practices as unsustainable.

**Conclusions:**

Financial constraints were identified by TB and MDR-TB patients and health care professionals as the most pervasive barrier to care. Barriers appeared to be magnified for ethnic minorities and patients coming from rural areas, especially those with MDR-TB. To reduce financial barriers and improve treatment outcomes, there is a need for further research into the total costs of seeking and accessing TB and MDR-TB care. This will enable better assessment and targeting of appropriate financial support for identified vulnerable groups and geographic development of relevant services.

**Electronic supplementary material:**

The online version of this article (doi:10.1186/s12889-017-4089-y) contains supplementary material, which is available to authorized users.

## Background

China has the second highest tuberculosis (TB) and multidrug-resistant TB (MDR-TB) burden in the world [1]. This is in spite of meeting its TB control targets, reducing overall prevalence and achieving 100% population coverage with free standardised TB services under Directly Observed Therapy Short Course (DOTS) [2]. However, in practice, service coverage is not equally distributed, with a higher concentration of resources in eastern China and urban areas [3]. Patients thus continue to face barriers to diagnosis, treatment and care, resulting in long delays in access to services and inequities in population burden of TB and accessing care [4–6]. The most commonly cited barrier to the successful treatment of both tuberculosis and multidrug-resistant tuberculosis (TB/MDR-TB) is financial [5–11]. Studies indicate that provision of free TB diagnosis and treatment mitigates direct costs, but does not eliminate indirect out-of-pocket patient costs [8, 12–16], which can be substantial and may include extra diagnostic tests, hospitalisation fees and drugs for side-effects [11, 12, 17–20]. These issues are likely to be further exacerbated for MDR-TB patients, due to the relatively high cost and complexity of care relative to drug sensitive TB, and those living rural areas [13, 21], who account for 70% of TB patients in China [22–24]. The New Rural Cooperative Medical Scheme (NRCMS) goes some way to alleviating the direct health-related costs for rural registered TB/MDR-TB patients. However, it does not cover outpatients costs, which often constitute the majority of expenses associated for TB/MDR-TB patients [25, 26]. Furthermore, patients living in remote and rural areas have to travel further and incur greater total costs for equivalent services, or may receive health care from providers with less experience and knowledge of TB [3, 5].

Total indirect and direct treatment costs associated with TB/MDR-TB care may equal or exceed a patient and/or household’s income-often identified as catastrophic health costs. Thus, patients may have to rely on a number of coping mechanisms (e.g. borrowing money, loans and selling assets) in an attempt to offset the costs of accessing care, which can push them into debt and poverty [7, 21, 27–35]. These financial barriers may be exacerbated for vulnerable population subgroups [36], including the less-educated, the elderly, ethnic minorities, farmers, migrant workers [20, 37, 38], those with co-morbidities such as diabetes [39, 40] and as well as varying with gender [41–45]. Such groups may be exposed to greater risk of developing disease, as well as worse treatment outcomes due to poor socioeconomic status, access, knowledge of TB and relevant services, lack of support and stigma [7, 11, 38, 41, 46, 47].

In this study, we explore barriers to successful care with a focus on MDR-TB patients, a population that has been relatively understudied to date [48]. In particular, we investigate how certain groups may face greater challenges to accessing TB services, the added challenges for patients with drug resistant disease, and how patients and healthcare providers cope in Yunnan Province, China. In particular, we pay attention to rural-urban differences and capture perspectives of TB/MDR-TB patients and frontline healthcare providers.

## Methods

### Study setting: Yunnan province and its TB control system

**Fig. 1:**
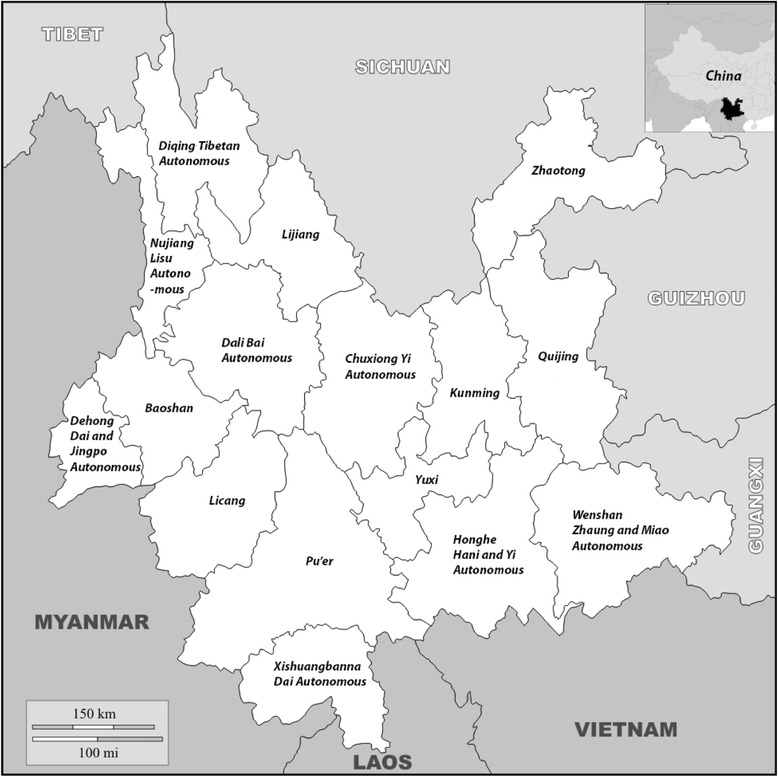
Map of Yunnan province and neighbouring countries. Map modified following terms and conditions of use (http://d-maps.com/conditions.php?lang=en) in accordance with ‘*D-Maps.com. Free maps, free blank maps, free outline maps, free base maps*’ copyright permissions: China (http://d-maps.com/carte.php?num_car=11570&lang=en) and Yunnan province (http://d-maps.com/carte.php?num_car=19490&lang=en)

Yunnan is China’s most south-westerly province (see Fig. 1) with a total area of 394,100 Km^2^, 94% of which is mountainous. Approximately 74% of its 46 million population are reported to live in rural areas and, although 67% belong to the Han ethnic group, Yunnan is one of China’s most ethnically diverse provinces. Compared to many other parts of the country, Yunnan has a relatively high TB burden (a provincial TB notification rate of 60 per 100,000) and poverty rate, and there is considerable geographic variation in TB burden (with prevalence rates as high as 1050 per 100,000 in some counties) and treatment outcomes across the province [6, 49].

Yunnan reported full DOTS coverage in 2004 and, by 2005, reported over 70% of smear-positive cases were being diagnosed through the DOTS programme and an average treatment success rate of over 90% [6]. However, substantial delays from the onset of symptoms to TB treatment have been recorded, with one third of patients delaying more than 90 days before seeking care at a DOTS-implementing health centre for their TB disease symptoms [5]. Furthermore, the cure rate was below 85% in 26 out of 129 counties in Yunnan, and non-cure was associated with low income [6]. The public’s knowledge of TB prevention and control in Yunnan has been reported as lower than national averages and that patients’ knowledge of TB was a significant factor in influencing treatment outcomes [6, 50].

Similar to other provinces across China, TB control is organised through a decentralised semi-vertical system [3, 9, 22], with more specialist skills and knowledge, activities and medical equipment found at higher administrative levels. All people with symptoms consistent with TB should be referred to the county TB dispensaries, which are affiliated with the Centre for Disease Control (CDC) and form the primary facilities for TB diagnosis and treatment. Yunnan’s ‘free’ TB treatment and diagnosis policy includes the following services: sputum smear test, chest X-ray and anti-TB medication distributed by the government [12]. Where other health services are required, patients may be referred as inpatients to the Tuberculosis Control Centre (TCC) or No. 3 Hospital, both located in Kunming.

While TB medication and diagnosis remain free at CDC facilities, free MDR-TB and extensively drug resistant TB (XDR-TB) treatment and diagnosis have been discontinued since June 2014 following the end of funding by The Global Fund to Fight AIDS, Tuberculosis and Malaria (GFATM). However, patients who commenced MDR-TB treatment before this date continue to receive free medication, together with some of the other GFATM funded care, which includes: free diagnosis; contributions to transport costs; and nutritional support. A second key difference between TB/MDR-TB treatment for rural patients, is that the former is decentralised to the county and village level, while the latter at the time of the study was centralised to Kunming (see Fig. 1).

### Study sampling and data collection

In-depth interviews and focus group discussions (FGD) were conducted between August 2014 and May 2015 with TB/MDR-TB patients (with a stronger emphasis on interviewing those diagnosed with MDR-TB where possible) and health professionals in the main TB hospitals in Kunming, as well as at health centres in two rural counties in Yunnan province. Sampling of participants and health centres in rural Kunming followed convenience and purposive approaches, with emphasis placed on exploring patients (Additional file 1 Annex 1) and health professionals’ (Additional file 2 Annex 2) diverse experiences and perceptions related to TB/MDR-TB, taking into account: gender, age, ethnicity, prefecture of residence, occupation, and TB/MDR-TB status. The limited number of health professionals working directly with TB/MDR-TB patients in the region, meant that data saturation (i.e. when novel ideas cease to emerge during recent interviews) was reached with a smaller sample of the former compared to the latter. Hence, FDGs were only conducted with patients, and were deemed unnecessary to explore health professionals accounts further.

The focus of the interviews and FGDs with patients (Additional file 3 Annex 3) was on their experiences, knowledge and perceptions of TB/MDR-TB, including: diagnosis, treatment and transmission; support and assistance; disclosure and impacts; and expectations for their future. While interviews with health professionals also covered many of these topics, they also explored perceptions of: barriers to care and treatment; stigma and social sanctions; roles of support networks; and lay understandings. Interviews varied in duration between 40 min to 3 hours (an hour and half on average), while FGDs lasted approximately one hour. The interviewer was a male native English speaker, while the interpreter was a female native Mandarin speaker. This allowed flexibility in terms of gender differences during interviews: i.e., if the participant felt more comfortable speaking to a woman they would direct their conversation to the interpreter or a man to the researcher.

### Data analysis

All in-depth interviews were conducted in Mandarin via simultaneous translation to English by a professional interpreter and digitally recorded, while the researcher took extensive field-notes. The FGDs were conducted in Mandarin and digitally recorded. The recordings were directly transcribed and translated to English (literal translation). Analysis followed a combination of open and thematic coding, based on consultation of previous literature and notes taken during fieldwork. Coding was conducted on FGD transcripts and a combination of field-notes and interview transcripts. Thus, codes were identified based on their recurrence and wider relevance, including those relevant to TB/MDR-TB literature. In addition, codes and preliminary findings were shared and discussed at three dedicated meetings with health professionals in Kunming, which provided further feedback on our interpretation of the data and allowed for any necessary fine-tuning of the analysis.

## Results

### Overview of study participants

Twenty-one interviews were conducted with health professionals (see Table 1); 13 of which, worked in specialist TB/MDR-TB wards or health centres. A total of 54 (see Table 2) patients participated, with 26 TB/MDR-TB patients (17 men and nine women) interviewed and an additional five FGDs conducted with four to eight patients in each (a total of 19 men and nine women); almost half of patients were confirmed with MDR-TB (25 in total, with a further 9 not sure of their status or waiting for confirmation). Women (44%) and men participated in almost equal numbers (see Table 2); an intentional effect of the purposive sample, which sought to provide a gender-balance to participants’ accounts. However, in actuality, health staff reported a lower number of women compared to men patients diagnosed and attending TB/MDR-TB services, which appeared to be evident, based on the difficulty in recruiting sufficient women into the study. All of the interviews and FGDs were conducted in Kunming city, except a total of eight patients and eight healthcare professionals, who were interviewed in their counties of residence and work-Luquan and Yiliang (over 120 and 50 km, respectively)-both in Kunming prefecture (see Fig. 1).

**Table 1:** Characteristics of healthcare providers interviewed (*n* = 21)

Position	Men	Women	Total
Doctor	4	6	10
Nurse	0	5	5
Outreach/Community worker	0	2	2
Management	1	3	4
Total	5	16	21

**Table 2:** Characteristics of patient interviewees and participants of FGDs (total = 54)

Participant’s characteristics	Women (*n* = 24)	Men (*n* = 30)
Age (years)		
18–30	7	10
31–45	8	6
46–60	8	12
60+	1	2
Ethnicity		
Han	17	24
Minority	7	6
Residence		
Kunming Prefecture (Yunnan Province)	8	8
Non-Kunming Prefecture (Yunnan Province)	13	19
Non-Yunnan Province	3	3
Urban/rural residence		
Urban	10	9
Rural	14	21
Occupation		
Farmer	8	18
Other	16	12
TB status		
TB	8	12
MDR-TB	12	13
Unknown/Waiting for drug-resistance test result	4	5

The predominance of farmers (48%) and rural patients (64%), as well as the presence of ethnic minorities (including Yi, Bai, Lisu, Lahu, Miao, and Zhuang) amongst those interviewed, appeared to reflect health staff’s observations, that patients with some or all of these characteristics were the most commonly and severely affected by TB/MDR-TB, and its associated costs. Farmers estimated their annual household incomes at up to 50,000 Yuan (approximately 8,000 USD). Interviews and FGDs also included: a construction contractor and workers, a banker, photographer, university students, a doctor, factory workers, a man who worked in human resources, a shop owner, a migrant worker, and retirees. The majority of patients came from (see Fig. 1) Yunnan prefectures other than Kunming (70%), such as Zhaotong, Diqing Tibetan Autonomous and Nujiang Lisu Autonomous prefectures, while a few came from provinces further away (i.e. Guizhou and Hunan provinces). Patients quoted costs of travel to Kunming ranging from 10 to 900 Yuan (1–140 USD) and up to 2 days of travel, including distances over 600 km and travelling parts of their journey on foot.

#### Financial barriers and inequalities in accessing TB/MDR-TB care

Healthcare providers and patients both described financial constraints as the most common challenge faced by patients; hence, we have elected to focus this paper’s analysis in relation to financial barriers. Others barriers discussed, many of which were also linked to socio-economic status, included: deterioration of a patient’s health, stigma, geographical accessibility to TB services, side effects, language, knowledge of TB and related health services, and lack of social and emotional support. Although most of the barriers were reported across gender, age, ethnicity, prefecture of residence, educational background and socioeconomic status, how they manifested, their impact on a patient’s treatment course, and the extent to which they were experienced varied according to these same factors.

### Financial burden in spite of “free” TB and MDR-TB treatment and diagnosis policy

Patients and staff viewed the availability of free TB and MDR-TB medication as central to ensuring adherence and treatment success. The majority of TB patients reported opting for the free TB medication provided at CDC treatment centres. However, a few said they paid for their medication at government hospitals quoting costs varying between 50 and 250 Yuan (7–39 USD) per month. The reasons they gave for paying for medication, included: did not trust free medication; side effects; and lack of awareness of its availability.

The impact of discontinuation of the GFATM funding for MDR-TB treatment and diagnosis was identified as a major financial challenge for all patients. Most MDR-TB patients struggled to provide the precise costs of their anti-MDR-TB drugs following the GFATM cuts. When given, the cost of MDR-TB exceeded those cited for TB medication, ranging from 400 to 10,000 Yuan (63 to 1,571 USD) per month. Even with the availability of free TB and MDR-TB services funded by the GFATM, patients would frequently cite paying 800–900 Yuan (125–140 USD) per month for ancillary drugs and, in some circumstances, were unable to differentiate between them and the anti-TB drugs.

### Costs of accessing care

Patients and staff described a disproportionate financial burden on rural groups and ethnic minorities, with higher total costs incurred due to longer distances travelled for relevant services and the greater impact of costs due to lower relative incomes and savings. In addition, a number of health staff noted that in specialist TB hospitals in Kunming it was not uncommon for TB tests for rural patients to be repeated due to a lack of trust in the reliability of tests conducted in their home counties, which contributed to further costs and delays for patients coming from outside Kunming. Healthcare providers also observed that ethnic minorities, who spoke their own languages and lacked fluency in Mandarin, would frequently incur extra costs related to their TB or MDR-TB care. This was evident in healthcare staffs’ reports and from ethnic minority patients interviewed; among the latter, all came from rural backgrounds and almost all were accompanied by at least one other family member, who would help care and translate for them during medical consultations, and which resulted in increased costs related to transport, accommodation and food. This was both observed and reported to be more common for ethnic minority women due to their relative lack of fluency in Mandarin compared to men.

For MDR-TB patients, there were additional barriers at the time of study, due to the centralisation of treatment and the duration of time (24 months) needed to complete it. All rural patients had to travel to Kunming on a monthly basis to receive their MDR-TB medication and any relevant care, further adding to costs of their treatment. For example, a Yi ethnic farming woman in her late forties, stated the following on travelling to Kunming to get her MDR-TB treatment:

First we take a motorbike to the closest village then we take a bus to the county capital, Chuxiong (see Fig. 1), it’s about 130 km and takes about four hours. I have the NRCMS, which pays about 50% […] It takes nine hours to get here to the hospital in Kunming and it costs about 150 Yuan (24 USD) for a single journey. We also have to stay in a guesthouse. The travel takes a long time and is costly. […] We have to come back every month to get the treatment. If two people come, we have to pay more than 100 Yuan (16 USD) each time. So while I go, my husband is in the house looking after the farm.

A senior female nurse working in a dedicated TB ward in Kunming provided a second example of one farming couple, both of whom had MDR-TB and were receiving support from the GFATM, she said:

[…] some patients have to walk several hours to get their daily injections, 6 months for MDR-TB, and 12 months for XDR-TB. […] And every month they have to come back here to pick up their medication […]

All the patients interviewed reported having some form of medical insurance and emphasised its importance in ameliorating the costs of TB/MDR-TB care; all rural dwelling patients and most of those whose parents were farmers, were covered by the NRCMS. Prices reported by patients for the insurance scheme ranged between 80 and 200 Yuan (12–31 USD) per year. Patients and healthcare professionals recognised that medical insurance went some way towards ameliorating the costs associated with TB/MDR-TB care. However, in all cases, payments were made on a reimbursement basis and hence accessing health services still required substantial upfront out-of-pocket payments. This meant that patients, especially those coming from rural areas, were presented with considerable financial challenges regardless of actual coverage levels. Options for accessing healthcare were more limited for MDR-TB patients, as medication and diagnostic tests could only be accessed in one designated hospital in Kunming; however, an additional hospital in Kunming was added as the study finished. As such, all MDR-TB patients from counties outside Kunming were forced to collect their medication in Kunming at a lower level of reimbursement.

#### Strategies for coping with financial barriers

Recognising that financial constraints continually emerged as the most significant barrier to care, we analysed patients’ and health staff’s coping strategies for mitigating and overcoming them.

### Individual and household financial resources

Patients reported using accumulated cash savings from previous years to pay for the costs associated with treatment, or selling household assets such as livestock and crops. It was not atypical for a patient from a farming background to report, ‘*We get our money selling our pigs and crops, and use our savings to pay for it*.’ However, especially for rural subsistence farmers, household savings based on income from selling their crops and piecemeal work, were typically reported to be insufficient to cover a full course of TB treatment, and even less so for MDR-TB. A few patients reported borrowing money from a financial institution to cover household costs, including their care.

### Direct and indirect family support

No patient stated that they were able to manage the costs of their MDR-TB or TB infection alone. Almost all said they relied in some capacity on their family, either for informal cash transfers in the form of gifts or loans, or for care giving as well as replacement labour. The MDR-TB infected Yi woman quoted earlier, stated:

I borrowed money from sisters and my husband’s sisters, but we still have not paid them all back yet. I only have 4000 Yuan (628 USD) and I still have to pay back one sister. We feel very uncomfortable. Our daughter has given us 1000 Yuan (157 USD) […]

A patient’s inability to work due to infection with TB or MDR-TB was frequently identified as a major burden for subsistence farmers, who had little disposable income. Under these circumstances, family members were also drawn upon to ensure the work on their farms did not stop. As a male farmer with TB said:

It affects our income. Once you are diagnosed with TB, you can’t work […] In the past, while I was healthy, my wife took care of the children, and I went out to work. Now, my wife goes out to work, and I stay home taking care of the children.

### Drawing on multiple coping strategies

Due to the extreme financial burden, most MDR-TB patients reported relying on multiple different sources of funding, having exhausted one after another over the long duration of treatment. A not uncommon example for rural residents and farmers was provided by a 41-year-old MDR-TB infected ethnic Lisu woman from remote north-western Diqing Tibetan Autonomous prefecture in Yunnan over 600 km from Kunming (see Fig. 1):

The first time I was hospitalised, we borrowed 6000 Yuan (942 USD). The second time we borrowed 5000 Yuan (785 USD). We have just paid our debts, and then we had to sell my motorbike for 4000 Yuan (628 USD) and borrow 8000 Yuan (1256 USD). We have borrowed money from our relatives; 1000 (157 USD) from one, another 1000 from others. Now we have to go back to sell the buffalos for about 4000–5000 Yuan each.

### Direct and indirect support by healthcare providers

Costs of accessing care were a major burden on patients, and could result in friction between some healthcare staff and patients. Staff in the specialist TB hospital in Kunming were well aware of the complexities and costs of TB/MDR-TB care and the burdens they placed on patients coming from rural and poorer areas. Providers and patients reported cases where providers made special efforts to support treatment. One MDR-TB infected man explained that his doctor had accommodated his need for a modified self-administered care plan, while continuing to prescribe the necessary drugs:

I live far from the clinic, about four to five kilometres by foot; it’s very poor access. Every time I got back from the clinic my coughing got worse. So I called the doctors and asked whether I could take the injections by myself, because the previous practice was very bad for me. The doctor said it was fine on the condition that I use the medicine subscribed by them. So I bought saline and bags by myself, and used the drug given by the doctor. After 1 month, my situation got a lot better.

Some healthcare providers also felt compelled to give directly via personal donations of clothes, money, odd jobs or surplus drugs. One nurse in her thirties working in Kunming noted:

Some people don’t even have enough money for food. One man came with his wife who helped to look after him. They had only one bun for each meal and one meal a day. So we gave them some money and free milk to help them […] when we gave them some medication we used stuff that hadn’t been completely finished (for example, when injections are done by weight).

In another case, a female nurse, who was largely responsible for counselling TB patients and their families, recounted how the providers at the TB hospital not only donated cash, but found ways to provide employment for the patients and their family members:

The mother and two sons have MDR-TB, so only the father could work to get money in Qujing prefecture (approximately 150 km east of Kunming; see Fig. 1), so for example, we thought of having her son as a peer educator and the father as doing some jobs in the clinic.

#### Impact on patients and healthcare providers

In spite of the presence of free or subsidised care, insurance and the supporting strategies adopted above, most patients and staff viewed TB/MDR-TB as having a catastrophic impact on household finances.

### Impoverishment

According to many healthcare providers and patients, it was not uncommon for patients to face impoverishment and the exhaustion of all financial reserves. This was described by a female farmer in her fifties with MDR-TB, from a county outside Kunming city (approximately 120 km.), when describing the situation of her MDR-TB infected husband:

Because he has diabetes, it has pushed up the costs a huge amount! Also he is unable to work, so the financial costs are very hard; treating TB and diabetes at the same time. We have had to borrow money from relatives. In recent years we have spent tens of thousands of Yuan. We have no savings left, we have used them all up!

At the same time, patients reported that the financial consequences resulted in reduced consumption or compromises in medication in ways that might adversely impact treatment success. In the case of the MDR-TB couple who were previously described as eating only one meal per day, another nurse described how the household still could not prevent bankruptcy, even while reducing consumption to near-starvation levels:

They live in a county that belongs to Kunming, in a very remote village that takes 2 days to get here. Only his check-ups and MDR-TB medication are covered by the GFATM, but not the transport or food. He has been on and off treatment for over 10 years. He even went bankrupt.

### Strained relationships and social stigma

Reliance on family or community members for support led in a number of cases to the erosion of social standing, strained relationships and social stigma. On a few occasions support was accompanied by serious questions as to the value of pursuing a course of medication and all the associated costs, when there was no guarantee whether they would be cured or not. This was particularly true for MDR-TB patients whose known length of infection and course of treatment was considerably longer than those with TB.

Patients often reported experiencing strong guilt and shame (as being a ‘burden’) when discussing the support, which they received from family members, relatives and friends. In addition to its physical toll, TB infection (and MDR-TB especially) eroded social capital, including affecting traditional gender and generational roles within a family, leading to social stigma and considerable pressure on their psychological well-being. This was noted by various MDR-TB patients, such as the two male farmers quoted below:

I feel indebted to my family, especially the children, I feel pressured. Because I am a farmer, I don’t have much income, now I have no income and feel guilty for my family. I have three children, the eldest one is in grade one of junior high school, another one is in grade five of primary school. They are all in school, we are short of money.

I feel so sad. I am sick and unable to help my family. Seeing my parents working, I feel so uncomfortable.

For many of the male patients, this manifested as a result of the disruption of their traditional role as a ‘bread earner’ and meant their wives, parents or other family members would potentially have to take on this role, leaving some men to taken on their wife’s role as a carer for their children. In another, perhaps extreme, case, an elderly woman with MDR-TB said that after borrowing money a number of times from her sisters for treatment, they had cut ties with her after they had repeatedly referred to her as useless and that she was better off dying, with which she herself agreed with:

They say “we hope you die.” They think I am a waste of money. […] So when my sisters complain about it, I respond, “I will just wait till my daughter is married. I don’t want to live or be treated after that.”

From a provider perspective, staff were well aware that helping patients beyond their remit was of limited use and that more systemic solutions would have to be found. As a nurse working in a TB designated centre in Kunming explained:

Staff also donated money in the past, but this is not sustainable. So we have to rely on government policy to help these people.

## Discussion

This paper explores experiences and opinions of healthcare providers and patients in Yunnan province, China, focusing on financial-related barriers and inequalities in the provision of, and access to, MDR-TB/TB care. Unlike other studies on this topic, we also investigated coping strategies and their impacts on patients and households, which potentially require the support of policy makers.

Our study indicates that financial constraints were identified as the most pervasive barrier to care amongst patients and health care professionals. Barriers appeared to be magnified for ethnic minorities and patients coming from rural areas [7], especially for farmers and those infected with MDR-TB. Cuts in the GFATM financing resulted in the withdrawal of previously subsidised service, and costs of extra diagnostic tests, drugs for side-effects and travel, accumulate during the long course of treatment. Typical coping strategies documented in other studies [7, 10, 21, 28, 29, 33] – such as selling their crops and household assets, taking out loans and relying on family members to provide financial and non-financial support-were also represented. We also found that healthcare providers empathised with MDR-TB patients and understood the magnitude of the challenges they face, trying to help beyond their medical care remit where possible, by voluntarily and spontaneously contributing financial and non-financial aid. Our findings indicate that these coping measures were frequently inadequate from the perspective of both patients and providers [30]. Patients and families were unable to avert poverty and the total-and potentially catastrophic-costs of accessing care, while experiencing a significant social emotional and psychological toll, for some in the form of strained familial relationships and social stigma. Furthermore, front-line providers were limited in their capacity to provide coping strategies that could cover shortfalls in patients’ finances through unorthodox means.

This study provides further insights into factors driving the higher TB burden and slower declines in prevalence in rural compared to urban areas in China [24]. Rural patients appear to face lower availability and quality of care, but higher absolute as well as relative costs due to the need to travel and lower incomes, respectively, and have been shown to be more likely resort to coping strategies compared to urban populations and suffer catastrophic costs [27, 33]. This is compounded by the fact that socioeconomic advantages, such as access to jobs and educational opportunities, quality of roads and availability of frequent public transport, all favour urban residents [51]. In the case of MDR-TB treatment, rural-urban inequities were particularly severe as medication, specialist medical knowledge and equipment are limited to urban locations (the provincial capital, Kunming), but were not available at county hospitals or village clinics. Although less pronounced in the case of TB treatment, these inequities also perpetuate a disproportionate risk of developing MDR-TB in the future [52, 53].

From a policy perspective, our work confirms that China’s subsidised TB and-previously subsidised - MDR-TB treatment are perceived to be necessary, but insufficient for ensuring continued access to relevant care for patients [10–12, 16]. Similarly, the New Rural Cooperative Medical Scheme provides crucial, but insufficient coverage of treatment for many patients [14, 27, 54, 55]. Thus, our findings are consistent with previous work suggesting that vulnerable populations, such as rural residents have benefitted least from China’s economic development and health system reforms over the last 30 years and that current measures are insufficient to provide financial protection from the potentially catastrophic costs of TB/MDR care, especially for low income groups [56, 57]. A recent attempt to make MDR-TB more affordable for patients in China by limiting patients out-of-pocket spending to 10% was still found to be insufficient [58]. Together with this study and others [21, 27, 32, 59], we suggest a need for greater attention to the total direct and indirect costs to individuals and society.

Differential financial support and targeted investment for vulnerable groups, especially for those coming from rural areas, which takes into account indirect costs (i.e. transport, loss of work, language barriers and home care) will be essential to financially protect patients and households and improve health outcomes. The following measures might go some way to resolving the total costs faced by some TB/MDR-TB patients: increased insurance reimbursements and subsides for MDR-TB patients; formal prepayments schemes and multi-sectoral social protection networks for households, with investment beyond the health sector; geographically targeted investment and development to reduce inequities in access to equivalent financial and health services, such as community DOTs; and where possible, decentralisation of quality TB/MDR-TB services [5, 29, 31, 32, 60–62]. The successful selection and implementation of such policies and interventions could benefit from supplementary quantitative research, which documents the magnitude and central determinants of patient costs, including, demographically disaggregated data on indirect costs, economic contributions of patient coping strategies and costs of care relative to household incomes [21, 27, 33]. This would not only ensure better outcomes for cost mitigation and reduction of financial barriers for patients access to care, but also treatment adherence and monitoring of progress to the End TB strategy target of zero catastrophic costs [63]. In addition, further qualitative exploration of the social impacts (i.e. impoverishment, strained relationships and social stigma) of costs associated with TB/MDR-TB care would strengthen the development and implementation of any interventions based on quantitative findings through providing relevant contextual data to improve delivery and targeting.

The main limitation of our study is its sample size and restricted geographical focus to the provincial capital city of Kunming, two surrounding counties and public health facilities; largely, a practical question of time and costs to conduct the research. Attempts were made to overcome these issues by interviewing patients from rural settings that had arrived at the (urban) specialist health centres; this provided insights into those who have succeeded, to varying degrees, in accessing services in Kunming despite potential barriers, and provides important insights into the factors necessary for other patients to do so. However, the experiences of patients that were not able to, or chose not to come to Kunming were not captured. This means that the study should not be taken as representative of Yunnan as whole. Rather, the experiences and opinions of those interviewed has enabled the identification of areas of potential concern for policy and future research.

## Conclusion

Our study shows that barriers to care and adverse impacts of disease may be exacerbated for rural patients, and especially those infected with MDR-TB, as critical health services (including, medication and diagnostic tests) are concentrated in major cities and costs of care can be catastrophic for some households. Geographical and socioeconomic differences not only contribute to the unequal burden of MDR-TB (and TB) costs, but may also potentially affect its development and spread. Coping mechanisms-such as patients’ reliance on family members and healthcare workers provision of personal contributions to the poorest individuals-are helpful, but often unsustainable and insufficient, raising new challenges. To reduce financial barriers, improve treatment outcomes and minimise catastrophic health costs, there is a need for further research into the total direct and indirect costs of accessing TB/MDR-TB care and their social consequences. This will enable better assessment and targeting of appropriate financial support for identified vulnerable groups and geographic development of TB/MDR-TB services.

## Additional files

Additional file 1: Annex 1 Interview Guide - Patients undergoing treatment for TB. (DOCX 12 kb)
Additional file 2: Annex 2 Interview Guide - Health Professionals involved in treatment for TB. (DOCX 11 kb)
Additional file 3: Annex 3 Focus Group Discussion Guide - MDRTB-/TB patients. (DOCX 8 kb)

## Abbreviations

CDC: Centre for disease control
DOTS: Directly observed therapy short course
FGD: Focus group discussion
GFATM: The global fund to fight aids, tuberculosis and malaria
MDR-TB: Multidrug resistant TB
TB: Tuberculosis
XDR-TB: Extensively drug resistant TB
