# Supplementary material for: Financial barriers and coping strategies: a qualitative study of accessing multidrug-resistant tuberculosis and tuberculosis care in Yunnan, China
Source: BMC Public Health. 2017 Feb 22;17:221. doi: 10.1186/s12889-017-4089-y (PMC5320743; doi:10.1186/s12889-017-4089-y)
Supplement: Additional file 1: — Annex 1 Interview Guide - Patients undergoing treatment for TB. (DOCX 12 kb) [file 12889_2017_4089_MOESM1_ESM.docx]

**Interview Guide - Patients undergoing treatment for TB**

| **Date:** | **Patient id:** | **Interviewer:** |
| --- | --- | --- |
| **Interview start time:** | **Interview end time:** | **Ethnicity:** |
| **Gender:** | **Age:** | **Occupation:** |

| **1.** | **Knowledge and beliefs about TB before and after diagnosis** |
| --- | --- |
|  | Thinking back, what did you know about tuberculosis before you were diagnosed?  *Prompts:* *Signs and symptoms, causes, transmission, treatment, prognosis, sources of knowledge* |
|  | Compared to before, do you think differently about tuberculosis now? - In what ways?  *Prompts:* *Signs and symptoms, causes, transmission, treatment, prognosis*  - Why this change?  *Prompts:* *Experiences, sources of knowledge* |
|  | How would you describe tuberculosis to someone who had not heard of it before?  *Prompts:* *Signs and symptoms, causes, transmission, treatment, prognosis, similarities and differences to other illnesses* |
|  | What advice would you give to someone who thought s/he might have tuberculosis?  *Prompts: What should they do? What kind{s} of treatment should they have?*  - Would your advice vary according to the person’s characteristics and situation?  *Prompts:* *Ethnicity, gender, age, marital status, employment status, children* |
|  | How do you think you came to have tuberculosis?  *Prompts: Susceptibility, transmission, vulnerabilities* |
|  |  |
| **2** | **Experience of TB symptoms from onset** |
|  | Thinking back to before your diagnosis with tuberculosis, what were the signs that you were unwell?  *Prompts: initial symptoms, change over time* |
|  | How did your day-to-day routine change when you began to feel unwell?  *Prompts: Impacts on employment outside the home, domestic and care work within the home, social roles, relations, and interactions, general wellbeing* |
|  |  |
| **3.** | **Route to diagnosis and treatment** |
|  | Beginning with the first, could you tell me about any treatments or medicines you tried after you felt unwell and before you were diagnosed with tuberculosis?  *Prompts: Try home remedies? Traditional medicines? Informally procured pharmaceuticals? What were their effects? Did you seek advice from anyone? – Who?*  - Is this what you would usually do if unwell?  *Prompts: How does it differ? Why is it different?* |
|  | How long were you unwell before you went to the clinic / hospital to be tested for tuberculosis? – Why then?  *Prompts: No improvement / deterioration of condition? Exhausted other options? Advice from trusted other? – Who? Removal of constraints – financial / social?* |
|  | Can you describe any difficulties or delays you faced in going to the clinic / hospital to be tested for tuberculosis?  *Prompts: What? How did you overcome them? What would help / hinder?* |
|  | Do you think most people would face the same difficulties or delays in going to the clinic / hospital to be tested for tuberculosis?  *Prompts: Why / why not? What type{s} of people face the biggest difficulties / delays?* |
|  |  |
| **4.** | **Experience of formal TB treatment** |
|  | Could you explain what this current treatment for tuberculosis involves?  *Prompts: Medicines, treatment process and duration* |
|  | How does this current treatment compare with any other solutions you tried in the past and / or are still trying?  *Prompts: Medicines, treatment process and duration, effects, expense*? |
|  | How has your day-to-day routine changed since you first began formal treatment for tuberculosis?  *Prompts: Impacts on employment outside the home, domestic and care work within the home, social roles, relations, and interactions, general wellbeing* |
|  | Can you describe any difficulties or delays you have faced in maintaining your treatment?  *Prompts: What? How did you overcome them? What would help / hinder?* |
|  | Do you think most people would face the same difficulties or delays in maintaining treatment?  *Prompts: How might they differ? What types of people face the biggest difficulties?* |
|  |  |
| **5.** | **Types and extent of assistance provided by friends, family, neighbours, health workers** |
|  | Can you describe any help you have received since you began to feel unwell?  *Prompts: Help with work inside or outside the home {including caring roles}, nursing through illness, income supplements or substitution {cash or in kind} loans / gifts?, help with travel to hospitals / clinics* |
|  | Can you describe any help you have received since you began treatment for tuberculosis?  *Prompts: Help with work inside or outside the home {including caring roles}, nursing through illness, income supplements or substitution {cash or in kind} loans / gifts?, help with travel to hospitals / clinics* |
|  |  |
| **6.** | **Disclosure** |
|  | Who, if anyone, can you talk freely about your illness with?  *Prompts: Why / Why not? Impacts of disclosure / discovery* |
|  | Who, if anyone, wouldn’t you want to know about your illness?  *Prompts: Why / Why not? Impacts of disclosure / discovery* |
|  | Would you say people are treated differently when it is known or thought they have tuberculosis?  *Prompts: By whom? Why do you think this? – experience? / word of mouth? What do you think accounts for this? What are the impacts?* |
|  | Would you say people feel differently about themselves once diagnosed with tuberculosis?  *Prompts: Why do you think this? – experience? / word of mouth? What do you think accounts for this? What are the impacts?* |
|  |  |
| **7.** | **Household / family impacts of TB illness and treatment** |
|  | Can you tell me about any impacts your illness / treatment have had on your family members?  *Prompts: changing roles within the household, proxy social sanctions* |
|  | Can you tell me about any impacts your illness or treatment has had on your relationships with your family members?  *Prompts: family members’ response to illness, impacts of any shifts in roles and relations* |
|  |  |
| **8.** | **Social impacts of TB illness and treatment** |
|  | Can you tell me about any impacts your illness or treatment has had on your relationships with your friends and neighbours?  *Prompts: changing roles and relations, social sanctions* |
|  |  |
| **9.** | **Income / expenditure impacts of TB illness and treatment** |
|  | Can you tell me about any impacts your illness or treatment has had on your income and spending?  *Prompts: impacts on income earning activities and opportunities, direct costs of securing treatment, indirect costs of securing treatment, loans, asset sales* |
|  |  |
| **10.** | **Expectations for future** |
|  | Do you think you will be able to complete the treatment programme?  *Prompts: Why / Why not? What would help / hinder?* |
|  | How do you imagine your life five years from now?  *Prompts: extent of recovery, legacy of illness, treatment, and any disclosure / discovery, type{s} of work undertaken, household situation, children’s circumstances, family relationships, social relationships, general wellbeing* |
