# Supplementary material for: Financial barriers and coping strategies: a qualitative study of accessing multidrug-resistant tuberculosis and tuberculosis care in Yunnan, China
Source: BMC Public Health. 2017 Feb 22;17:221. doi: 10.1186/s12889-017-4089-y (PMC5320743; doi:10.1186/s12889-017-4089-y)
Supplement: Additional file 2: — Annex 2 Interview Guide - Health Professionals involved in treatment for TB. (DOCX 11 kb) [file 12889_2017_4089_MOESM2_ESM.docx]

**Interview Guide – Health Professionals involved in treatment for TB**

| **Date:** | **Interviewee id:** | **Interviewer:** |
| --- | --- | --- |
| **Interview start time:** | **Interview end time:** | **Ethnicity:** |
| **Gender:** | **Age:** | **Occupation:** |

| **1.** | **Professional experience and perceptions** |
| --- | --- |
|  | Could you begin by describing the kinds of work you do with TB patients?  *Prompts: routine, ad hoc* |
|  | What would you say are the key impacts of TB illness, diagnosis and treatment on your patients?  *Prompts: Daily lives, livelihoods, roles, relationships, and self-perceptions* |
|  | In what ways – if any – do the key impacts of TB illness, diagnosis and treatment vary according to patients’ characteristics and situation?  *Prompts: Ethnicity, gender, age, marital status, employment status, children* |
|  |  |
| **2.** | **Perceptions of lay understandings** |
|  | What do patients typically know or believe about TB and TB treatment when you first meet them?  *Prompts: Signs and symptoms, causes, transmission, treatment regime /duration / protocol, prognosis*  – What are the impacts of these beliefs?  *Prompts: use of prior / concurrent treatments, response to diagnosis, attitudes to treatment, self-perceptions, disclosure* |
|  | In what ways – if any - do patients’ beliefs about TB and TB treatment vary according to their characteristics and situation?  *Prompts: Ethnicity, gender, age, marital status, employment status, children* |
|  | How would you say TB is typically perceived by the wider community?  *Prompts: Signs and symptoms, causes, transmission, treatment regime /duration / protocol, prognosis*  – What are the impacts of these perceptions? |
|  | How would you describe tuberculosis to someone who had not heard of it before?  *Prompts:* *Signs and symptoms, causes, transmission, treatment, prognosis, similarities and differences to other illnesses* |
|  | How do your friends and families view your work with tuberculosis patients? |
|  |  |
| **3.** | **Barriers to treatment and cure** |
|  | What kinds of obstacles do patients face in accessing and successfully completing TB treatment?  *Prompts: health, logistical, financial, social, familial* |
|  | In what ways – if any – do such obstacles vary according to the patient’s characteristics and situation?  *Prompts: Ethnicity, gender, age, marital status, employment status, children* |
|  | Based on your experience, how do you think these obstacles could be successfully addressed? |
|  | What – if anything – would you say characterises patients who struggle or fail to maintain treatment?  *Prompts: their characteristics, their household / family characteristics, their broader health, their experience of treatment seeking, healthcare provisioning* |
|  |  |
| **4.** | **Perceived roles of patient support networks** |
|  | How important would you say support from family and friends are for patient outcomes?  *Prompts: Help with work inside or outside the home {including caring roles}, nursing through illness, income supplements or substitution {cash or in kind} loans / gifts?, help with travel to hospitals / clinics* |
|  | How important would you say support from medical professionals and health workers are for patient outcomes?  *Prompts: Knowledge provision, moral support, logistical assistance, advice* |
|  | In what ways do TB and TB treatment impact patient’s family members and family relationships?  *Prompts: family members’ response to illness, impacts of any shifts in roles and relations, family members’ experience of proxy social sanctions* |
|  |  |
| **5.** | **Perceptions of stigma and social sanctions** |
|  | Would you say that patients feel able to speak freely of their condition?  *Prompts: Why / Why not? With whom? - Family, friends, neighbours, co-workers, employers, health professionals?* |
|  | In your experience, what kinds of social and / or economic sanctions – if any - do patients face if their condition is disclosed / discovered? |
|  | In what ways – if any – do social sanctions and stigma vary by patient’s characteristics and situation?  *Prompts: Ethnicity, gender, age, marital status, employment status, children* |
|  | Have you ever encountered any hostility or stigma as a result of your work with tuberculosis patients?  – Could you describe the circumstances?  *Prompts: From whom? Frequency? Duration? Impacts?* |
|  |  |
| **6.** | **Changes and recommendations** |
|  | Which aspects of your work – if any – would you like to see changed, if possible? |
|  | Which aspects of TB treatment more generally – if any – would you like to see changed, if possible? |
|  | Are there any other aspects of TB or TB treatment that you would like to discuss? |

| **7.** | **Compensation and incentives** |
| --- | --- |
|  | How much does a typical health worker in your area receive in salary, including annual bonuses? Are salaries consistently paid? |
|  | Is it more or less costly and/or time consuming to provide care to TB patients, compared to other types of patients?  *Prompts: Home visits?* |
|  | Do you receive any compensation, benefits or other incentives for treating TB patients, or other kinds of patients?  *Prompts: Travel allowances? Additional payments for working in rural areas? from whom?* |
|  | Do salaries and other working conditions vary across types of healthcare organisations?  *Prompts: NGOs versus government versus private?* |
