# Supplementary material for: Financial barriers and coping strategies: a qualitative study of accessing multidrug-resistant tuberculosis and tuberculosis care in Yunnan, China
Source: BMC Public Health. 2017 Feb 22;17:221. doi: 10.1186/s12889-017-4089-y (PMC5320743; doi:10.1186/s12889-017-4089-y)
Supplement: Additional file 3: — Annex 3 Focus Group Discussion Guide - MDRTB-/TB patients. (DOCX 8 kb) [file 12889_2017_4089_MOESM3_ESM.docx]

**Focus Group Discussion Guide – MDR-TB/TB patients**

The focus group discussions will examine MDR/TB patients experiences of TB/MDR and relevant care.

1. **What did you know about tuberculosis before you were diagnosed?**
2. **What advice would you give to someone who thought s/he might have tuberculosis?**
3. **Who, if anyone, can you talk freely about your illness with? / Who, if anyone, wouldn’t you want to know about your illness? Why?**
4. **Would you say people are treated differently when it is known or thought they have tuberculosis? How? Why?**
5. **Would you say people feel differently about themselves once diagnosed with tuberculosis? How? Why?**
6. **Can you tell me about any impacts your illness / treatment have had on your family members? Friends? Work? Studies?**
7. **How has tuberculosis affected your plans for your future?**
